# Supplementary material for: A causal framework for evaluating drivers of policy effect heterogeneity using difference-in-differences
Source: Health Serv Outcomes Res Methodol. 2025 Oct 25;26(1):26–47. doi: 10.1007/s10742-025-00358-5 (PMC12967569; doi:10.1007/s10742-025-00358-5)
Supplement: Supplementary file 1 — (pdf 58 KB) [file 10742_2025_358_MOESM1_ESM.pdf]

Supplemental Material for "A Causal Framework for Evaluating Drivers of Policy Effect Heterogeneity Using Difference-in-Differences"

by Gary Hettinger, Youjin Lee, and Nandita Mitra

Web Appendix A: Efficient Influence Functions

The efficient influence function (EIF) for the ATT (Sant'Anna and Zhao, 2020) is given as:

$$\phi^{(ATT)}(\mathbf{X}, A, Y_0, Y_1) = \frac{A - \pi_A(\mathbf{X})}{P(A=1)(1-\pi_A(\mathbf{X}))}(Y_1 - Y_0 - \mu_{0,\Delta}(\mathbf{X}))$$

The EIF for the $ADUTT$ (Hettinger et al., 2025) is given as:

$$\phi^{(ADUTT)}(\mathbf{X}, A, \mathbf{D}, Y_0, Y_1) = \frac{A}{P(A=1)}\xi(\mathbf{X}, A, \mathbf{D}, Y_0, Y_1) - \tau(\mathbf{X}, A, Y_0, Y_1) + J(\mathbf{X}, A)$$

Where

$$\xi(\mathbf{X}, A, \mathbf{D}, Y_0, Y_1; \mu_{1,\Delta}, \pi_D) = m(\mathbf{D}|A=1) + \frac{(Y_1 - Y_0) - \mu_{1,\Delta}(\mathbf{X}, \mathbf{D})}{\pi_D(\mathbf{X}, \mathbf{D})}P(\mathbf{D}|A=1)$$

$$\tau(\mathbf{X}, A, Y_0, Y_1; \mu_{0,\Delta}, \pi_A) = \frac{A}{P(A=1)}\mu_{0,\Delta}(\mathbf{X}) + \frac{(1-A)\pi_A(\mathbf{X})[(Y_1 - Y_0) - \mu_{0,\Delta}(\mathbf{X})]}{P(A=1)(1-\pi_A(\mathbf{X}))}$$

$$J(\mathbf{X}, A; \mu_{1,\Delta}) = \frac{A}{P(A=1)}\int_{\mathbb{D}}\{\mu_{1,\Delta}(\boldsymbol{\delta}, \mathbf{X}) - m(\boldsymbol{\delta}|A=1)\}dP(\boldsymbol{\delta}|A=1)$$

$$m(\mathbf{D}|A=1) = \int_{\mathbb{X}}\mu_{1,\Delta}(\mathbf{x}, \mathbf{D})dP(\mathbf{x}|A=1)$$

$$P(\mathbf{D}|A=1) = \int_{\mathbb{X}}\pi_D(\mathbf{x}, \mathbf{D})dP(\mathbf{x}|A=1)$$

Notably, the EIF for the ADT is not tractable as this functional is not pathwise differentiable without imposing parametric assumptions on the curve itself (Kennedy, 2024). However, the EIF for the ADUTT can be used to robustly estimate the ADT (Hettinger et al., 2025). As alluded to in the main text, $J(\mathbf{X}, A)$ does not factor into point estimates for our estimands of interest.

Web Appendix B: Block Bootstrapping Approach

Closed-form approaches are possible for ATT and $ADUTT$, but do not carry the robustness properties of the estimators since they rely on parametric assumptions violated under misspecified models. Sandwich variance estimators are a happy medium between the computation time of bootstrap approaches and robustness limitations of standard closed form solutions, but require new algebraic definitions for each set of estimating models, which are not always possible. Instead, bootstrap approaches generally maintain robustness and can be adapted for different models as well as spatial structures, albeit under potentially computationally intensive procedures. with the bootstrapping approach.

Once blocks are defined, our procedure works as follows:

1. Sample weights, γ_b, for each block $b = 1, \ldots, n_b$ from an independent distribution, i.e., $\gamma_b \sim$ exponential(1).

2. For each store i, sum all of the weights pertaining to store i as $\gamma_i = \sum_{b=1}^{n_b} \mathbb{1}\{i \in b\}\gamma_b$.

3. Normalize weights so the average weight within each treatment group is one, i.e., $\gamma_i = (\gamma_i \sum_{j=1}^{n} \mathbb{1}\{A_j = A_i\}) / \sum_{i=1}^{n} (\mathbb{1}\{A_j = A_i\}\gamma_i)$.

4. Plug weights into each step in the estimation process that relies on the sample.

   i. In estimation step (1), fit models for $\hat{\mu}_{1,\Delta}$, $\hat{\mu}_{0,\Delta}$, $\hat{\pi}_A$, and $\hat{\pi}_D$ with weights γ_i.

   ii. In estimation step (2), when calculating $m(\mathbf{D}|A = 1)$ and $P(\mathbf{D}|A = 1)$, use a weighted empirical average.

   (a) In estimation step (4), fit a weighted kernel regression with weights γ_i.

   (b) In estimation step (5), use weighted empirical averages for empirical means.

5. Repeat for each bootstrap sample and take the standard deviation (σ) of desired parameters for confidence intervals (i.e., add and subtract 1.96σ from point estimate for 95% CIs).

The weighted block-sampling design comes with several benefits. First, it addresses potential spatial correlation within blocks. Second, store-level weights are constant over time, thereby addressing temporal correlation within stores. Finally, by sampling continuous weights instead of discrete samples, this approach is more efficient for small samples by improving the observed support of $\mathbf{X}$ and $\mathbf{D}$ within given bootstrap samples.

References

Hettinger, G., Lee, Y., and Mitra, N. (2025). Multiply robust difference-in-differences estimation of causal effect curves for continuous exposures. *Biometrics* **81,**.

Kennedy, E. H. (2024). Semiparametric doubly robust targeted double machine learning: a review. In Laber, E., Chakraborty, B., Moodie, E. E. M., Cai, T., and van der Laan, M., editors, *Handbook of Statistical Methods for Precision Medicine*, pages 207–236. Chapman and Hall, New York, 1 edition.

Sant'Anna, P. H. and Zhao, J. (2020). Doubly robust difference-in-differences estimators. *Journal of Econometrics* **219,** 101–122.
